# Supplementary material for: BBX24 Increases Saline and Osmotic Tolerance through ABA Signaling in Arabidopsis Seeds
Source: Plants (Basel). 2023 Jun 21;12(13):2392. doi: 10.3390/plants12132392 (PMC10346558; doi:10.3390/plants12132392)
Supplement: Supplementary file 1 [file plants-12-02392-s001.zip › plants-2437945 Table S1.pdf]

Suppl. Table S1: P*rimers* used for selection of mutant lines and qPCR

Primes for PCR

Gen	Locus	Salk line	Primers
BBX24	AT1G06040	SALK_067473	Rp_TGAAAGAAATCGTCAACAGCC<br>Lp_ GACGAGAGGAAGAAGGAGAGC<br>LBa1_ TGGTTCACGTAGTGGGCCATCG
ABI5	AT2G36270	SALK_013163C	Rp_CACTCGTTTTCTTCTTAAAGCG<br>Lp_CAATGGAAGTTCGGAATCATG<br>LBb1.3_ ATTTTGCCGATTTCGGAAC
HY5	AT5G11260		For_TAAGAAAAATGCAGGAAC<br>Rev-WT_CTCATCGCTTTC AATTCC<br>Rev-215_CTCATCGCTTTC AATTCT

Primers for qPCR

Gene	Locus	Fw	Rv
BBX24	AT1G06040	AAGCCAGCAGCAACAACAACC	GAGAAGTGAAAGAAATCGTCAACAGC
ABI3	AT3G24650	CCGCCAACCTCGCAGTATAATC	CCTCCTCTGTCTCGCCATCC
ABI5	AT2G36270	CAATCAACAACAAGCAGCAGCAG	CCACCACTAAAGACACCAACACC
UBC	AT5G25760	CTGCGACTCAGGGAATCTTCTAA	TTGTGCCATTGAATTGAACCC
XERICO	AT2G04240	TGAGGTTTGAGAGCTTGTGCAGG	GAAAGCTGATGGTCTTCTGGCAC
PIF6	AT3G62090	ACAACAGTTTCTGCCTGCAC	GCATTGGCATTAGTCCAGGATG
