# Supplementary material for: BBX24 Increases Saline and Osmotic Tolerance through ABA Signaling in Arabidopsis Seeds
Source: Plants (Basel). 2023 Jun 21;12(13):2392. doi: 10.3390/plants12132392 (PMC10346558; doi:10.3390/plants12132392)
Supplement: Supplementary file 1 [file plants-12-02392-s001.zip › plants-2437945-supplementary.pptx]

## Slide 1
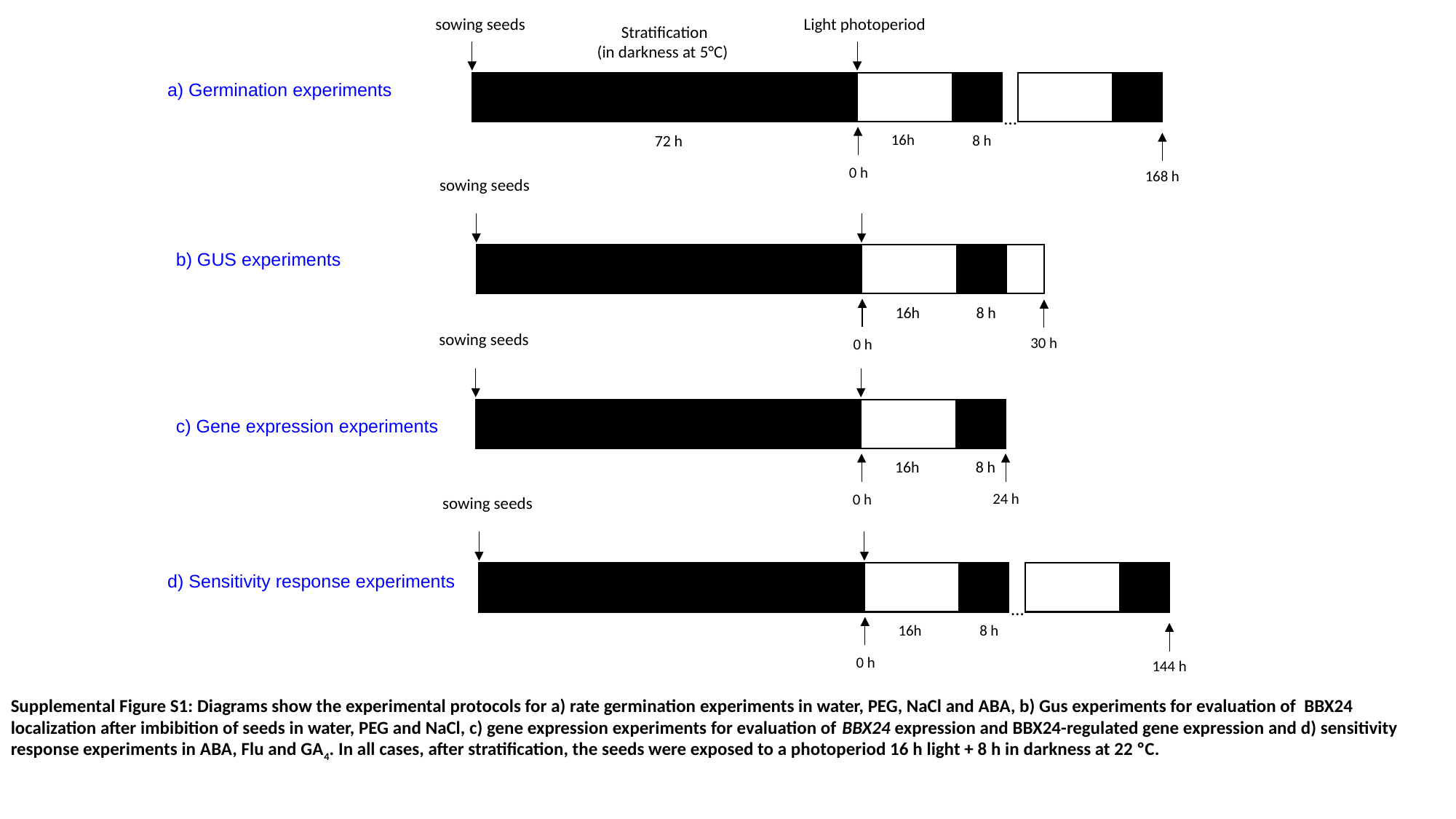

sowing seeds
Light photoperiod
Stratification
(in darkness at 5°C)
16h
8 h
72 h
…
0 h
168 h
a) Germination experiments
sowing seeds
16h
8 h
30 h
0 h
b) GUS experiments
sowing seeds
16h
8 h
24 h
0 h
c) Gene expression experiments
sowing seeds
16h
8 h
…
0 h
144 h
d) Sensitivity response experiments
Supplemental Figure S1: Diagrams show the experimental protocols for a) rate germination experiments in water, PEG, NaCl and ABA, b) Gus experiments for evaluation of BBX24 localization after imbibition of seeds in water, PEG and NaCl, c) gene expression experiments for evaluation of BBX24 expression and BBX24-regulated gene expression and d) sensitivity response experiments in ABA, Flu and GA4. In all cases, after stratification, the seeds were exposed to a photoperiod 16 h light + 8 h in darkness at 22 ºC.

## Slide 2
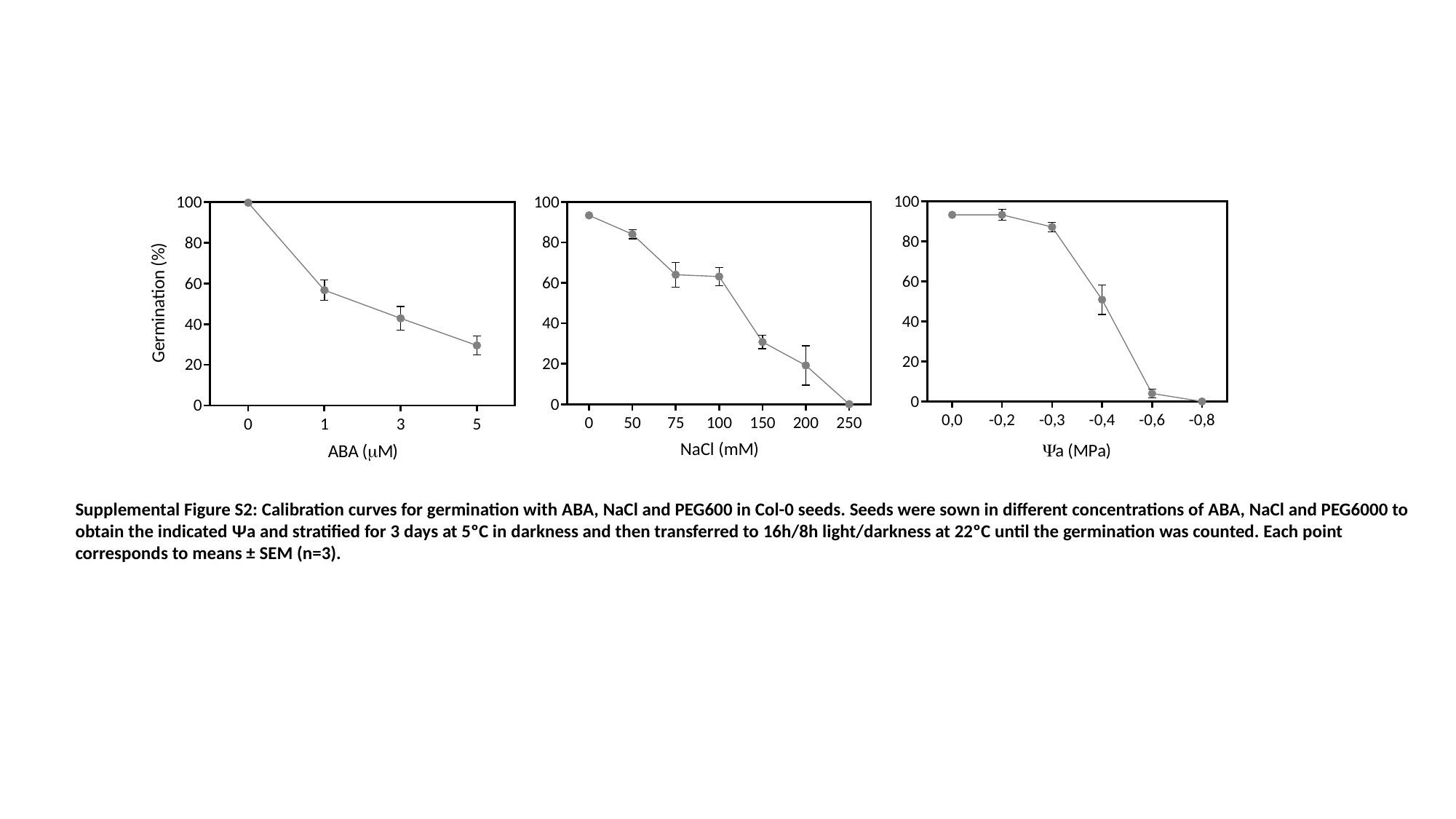

Supplemental Figure S2: Calibration curves for germination with ABA, NaCl and PEG600 in Col-0 seeds. Seeds were sown in different concentrations of ABA, NaCl and PEG6000 to obtain the indicated Ψa and stratified for 3 days at 5ºC in darkness and then transferred to 16h/8h light/darkness at 22ºC until the germination was counted. Each point corresponds to means ± SEM (n=3).

## Slide 3
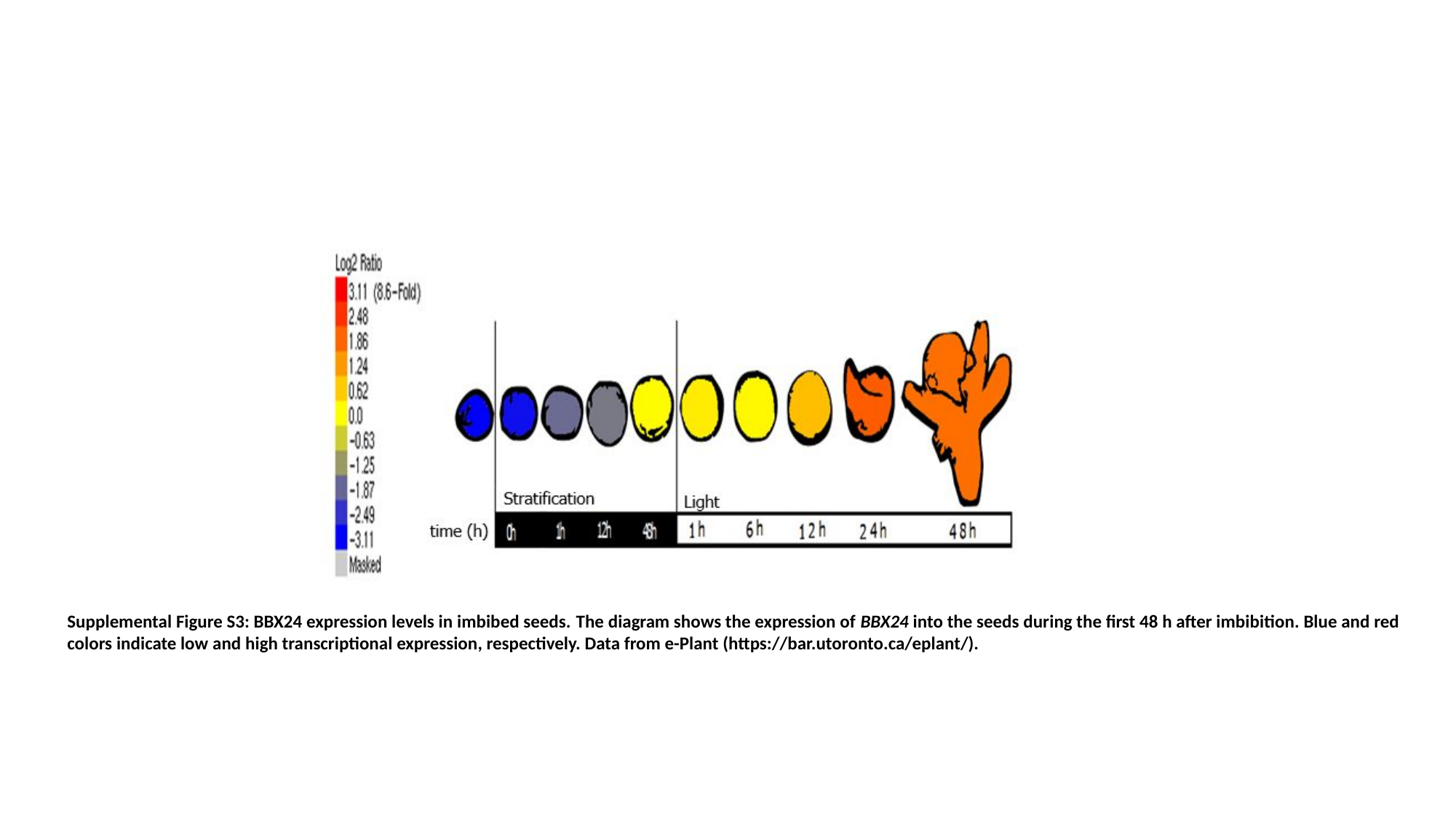

Supplemental Figure S3: BBX24 expression levels in imbibed seeds. The diagram shows the expression of BBX24 into the seeds during the first 48 h after imbibition. Blue and red colors indicate low and high transcriptional expression, respectively. Data from e-Plant (https://bar.utoronto.ca/eplant/).

## Slide 4
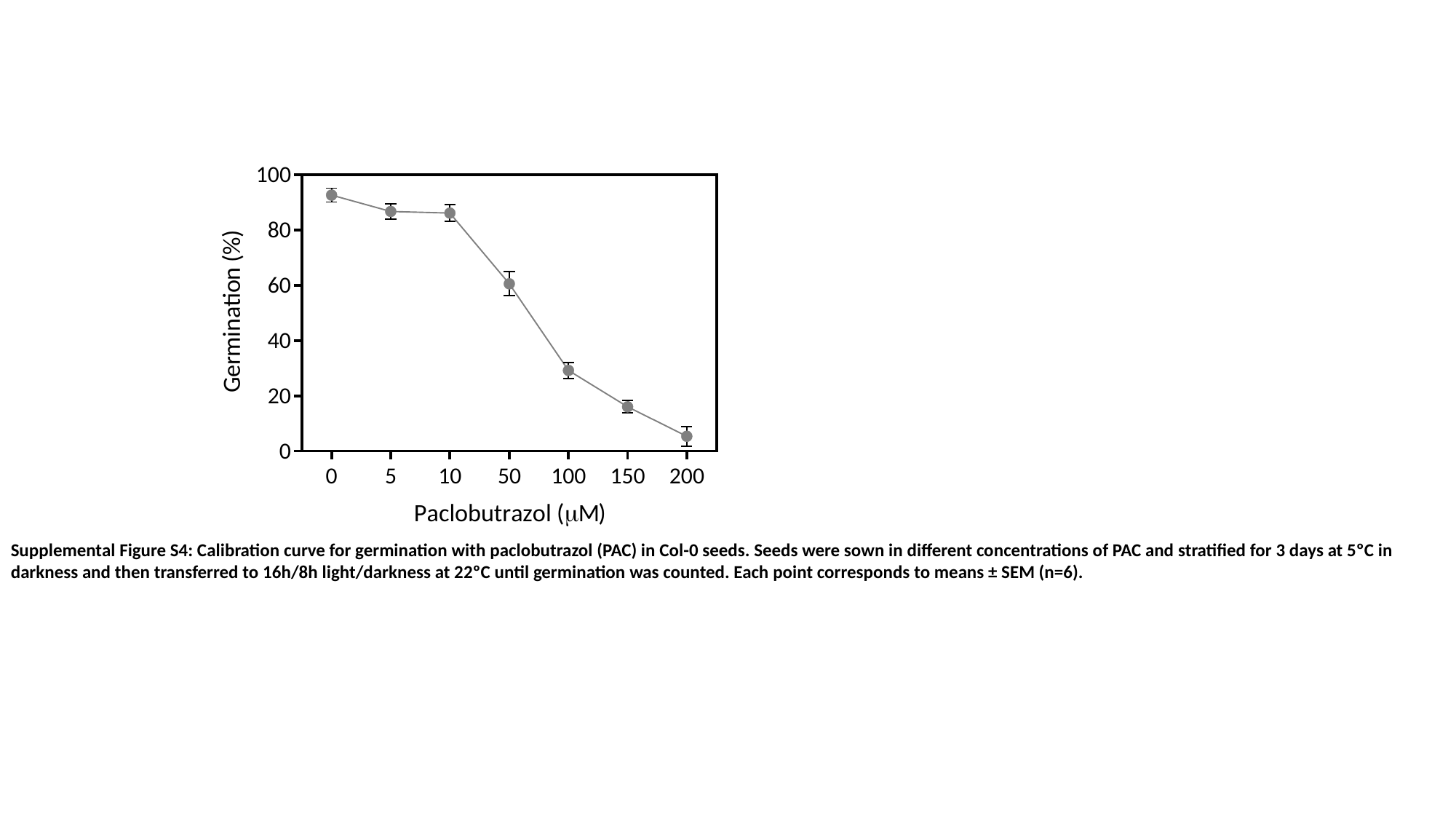

Supplemental Figure S4: Calibration curve for germination with paclobutrazol (PAC) in Col-0 seeds. Seeds were sown in different concentrations of PAC and stratified for 3 days at 5ºC in darkness and then transferred to 16h/8h light/darkness at 22ºC until germination was counted. Each point corresponds to means ± SEM (n=6).

## Slide 5
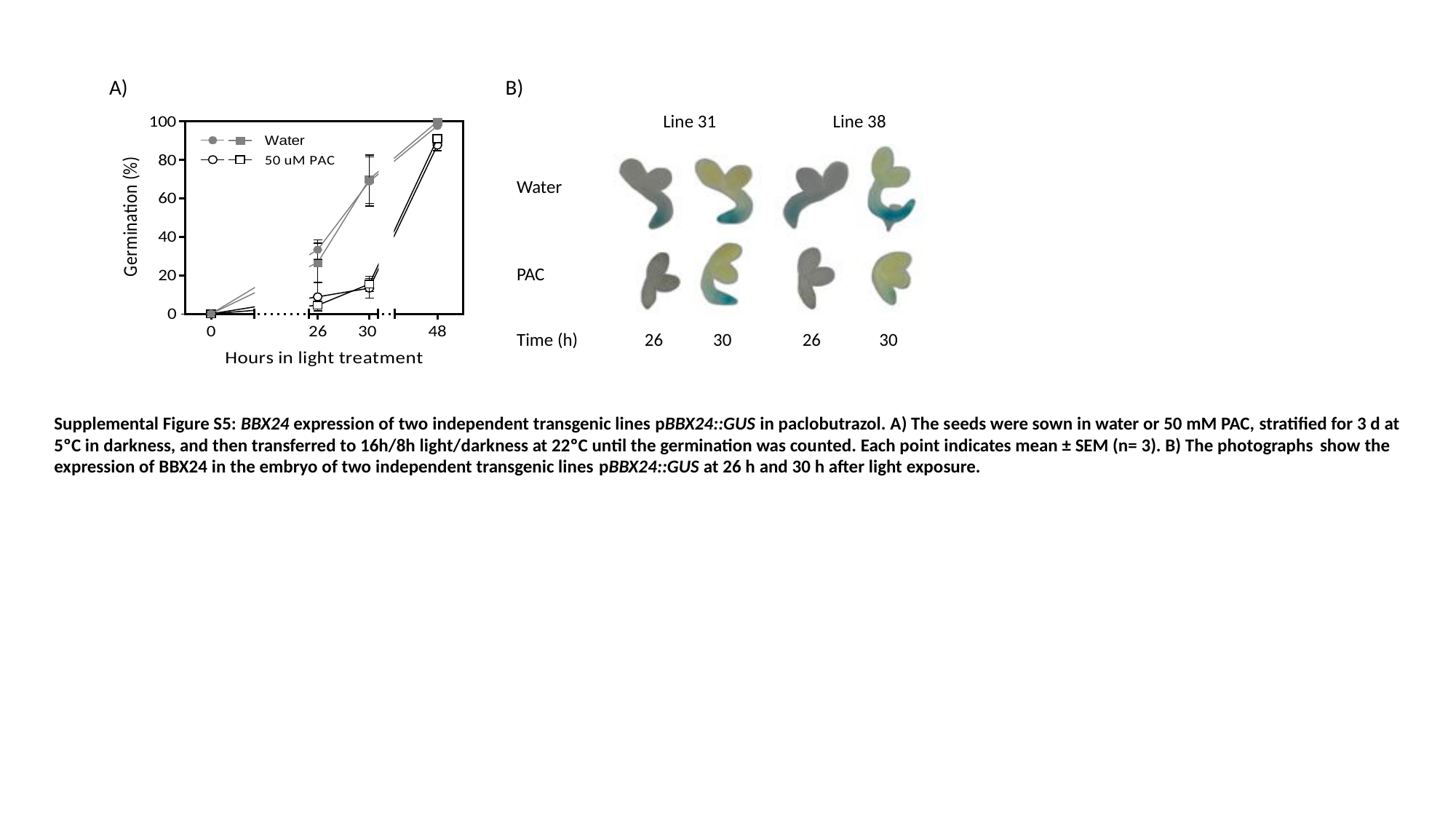

A) B)
 Line 31 Line 38
Water
PAC
Time (h) 26 30 26 30
Supplemental Figure S5: BBX24 expression of two independent transgenic lines pBBX24::GUS in paclobutrazol. A) The seeds were sown in water or 50 mM PAC, stratified for 3 d at 5ºC in darkness, and then transferred to 16h/8h light/darkness at 22ºC until the germination was counted. Each point indicates mean ± SEM (n= 3). B) The photographs show the expression of BBX24 in the embryo of two independent transgenic lines pBBX24::GUS at 26 h and 30 h after light exposure.

## Slide 6
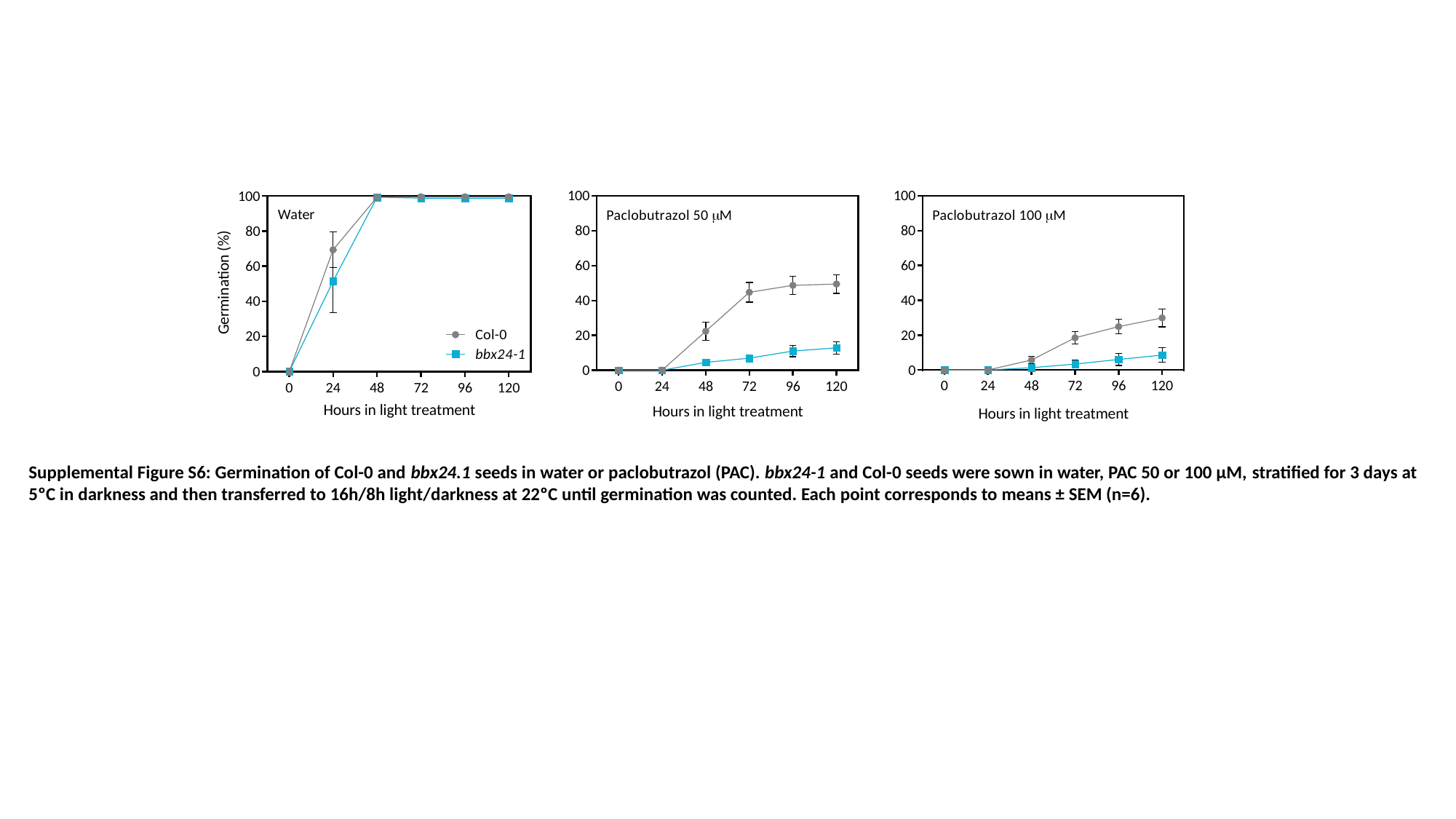

Supplemental Figure S6: Germination of Col-0 and bbx24.1 seeds in water or paclobutrazol (PAC). bbx24-1 and Col-0 seeds were sown in water, PAC 50 or 100 µM, stratified for 3 days at 5ºC in darkness and then transferred to 16h/8h light/darkness at 22ºC until germination was counted. Each point corresponds to means ± SEM (n=6).

## Slide 7
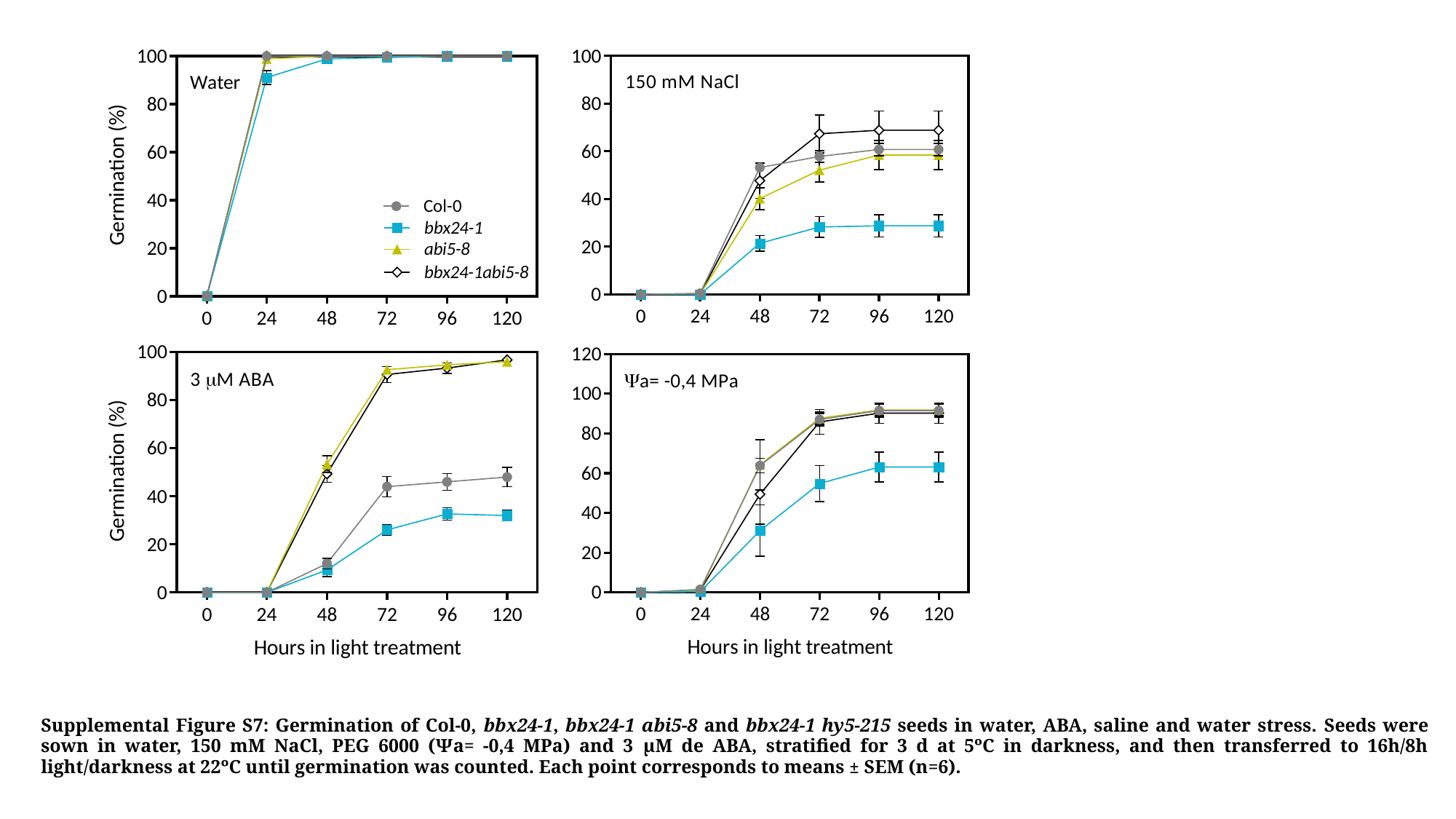

Supplemental Figure S7: Germination of Col-0, bbx24-1, bbx24-1 abi5-8 and bbx24-1 hy5-215 seeds in water, ABA, saline and water stress. Seeds were sown in water, 150 mM NaCl, PEG 6000 (Ψa= -0,4 MPa) and 3 µM de ABA, stratified for 3 d at 5ºC in darkness, and then transferred to 16h/8h light/darkness at 22ºC until germination was counted. Each point corresponds to means ± SEM (n=6).
